# Acute Arterial Thrombosis of Lower Extremities in COVID-19 Patients

**DOI:** 10.3390/jcm11061538

**Published:** 2022-03-11

**Authors:** Robert Glavinic, Ljiljana Marcic, Stipe Dumancic, Mirela Pavicic Ivelja, Irena Jeličić, Danijela Kalibovic Govorko, Ivana Medvedec Mikić

**Affiliations:** 1 Department of Infectious Diseases, University Hospital of Split, Soltanska 1, 21000 Split, Croatia; robert.glavinic@yahoo.com (R.G.); mmarendic@gmail.com (M.P.I.); irenajelicic80@gmail.com (I.J.); 2 Department of Radiology, Policlinic Medicol, Soltanska 1, 21000 Split, Croatia; lmarcic@mefst.hr; 3 University Department of Health Studies, University of Split, Rudera Boskovica 35, 21000 Split, Croatia; 4 School of Medicine, University of Split, Soltanska 2, 21000 Split, Croatia; stipe.dumancic@gmail.com (S.D.); dkalibovic@gmail.com (D.K.G.); 5 Department of Maxillofacial Surgery, University Hospital of Split, Soltanska 1, 21000 Split, Croatia

**Keywords:** SARS-CoV-2, COVID-19, arterial thrombosis, lower extremity, amputation, computed tomography angiography

## Abstract

Clinical signs and symptoms of COVID-19 varied from asymptomatic forms to severe, life-threatening conditions that required treatment in intensive care units. These severe forms of illness are connected with a hypercoagulable state due to excessive inflammation, hypoxia, immobilisation, and altered angiotensin-converting enzyme 2 (ACE-2). In total, 17 COVID-19 positive patients were diagnosed with peripheral arterial thrombosis (AT), 13 of them had COVID-19 pneumonia. Laboratory findings in patients with X-ray confirmed pneumonia showed a four times higher neutrophil-to-lymphocyte ratio (NLR), C-reactive protein (CRP) and three times higher lactate dehydrogenase level (LDH) than patients without confirmed pneumonia. Patients with pneumonia had significantly more bilateral occlusions of the lower extremities and a significantly higher percentage with complete occlusion of the arteries than patients without pneumonia. The rate of limb loss was 35.3%. They were all from the group with COVID-19 pneumonia. Ten out of thirteen patients with pneumonia died due to acute respiratory distress syndrome (ARDS). All patients without pneumonia were discharged from the hospital. The aim of this retrospective study was to report the incidence of arterial thrombosis of lower extremities and their complications in the acute phase of the infection among COVID-19 patients admitted to the hospital for treatment.

## 1. Introduction

The coronavirus (COVID-19) caused by the SARS-CoV-2 virus emerged in December 2019 in China and spread worldwide, causing the illness of more than one hundred million people. The virus has led to an increase in hospital admissions and is the leading cause of hospitalisation admittance to intensive care units, and holds a higher risk of mortality when pulmonary symptoms present, with respiratory distress as its most dangerous manifestation [1]. Accurate prediction of clinical outcomes for patients across this spectrum is often tricky. Cardiovascular and haematological involvement in COVID-19 is less well known, although more and more articles considering this problem are being published [2,3]. Clinical signs and symptoms of COVID-19 varied from asymptomatic forms to severe, life-threatening conditions that required treatment in intensive care units [4]. Ongoing reports concerning severely ill patients suggest that these severe forms are connected with a hypercoagulable state due to excessive inflammation, hypoxia, immobilisation, and altered angiotensin-converting enzyme 2 (ACE-2) [5,6]. Recent studies have shown that abnormal coagulation dysfunction is associated with a poor prognosis in patients with COVID-19 [7]. Laboratory findings in severe COVID-19 patients support these findings and show an elevation in D-dimer, fibrinogen, and prothrombin levels. Proinflammatory cells, such as interleukin (IL)-6, tumour necrosis factor, and CRP (C-reactive protein) are elevated [8]. LDH (lactate dehydrogenase) proved to be a prognostic COVID-19 pneumonia parameter: higher values point to more severe pneumonia. The neutrophil/lymphocyte ratio (NLR) presents the number of neutrophils divided by the number of lymphocytes. The rise in a neutrophil count represents a systemic inflammatory process, while a decrease in lymphocytes demonstrates ongoing stress inflicted by the disease [9]. The risk of AT (arterial thrombosis) may increase if the severity of pneumonia is high, including the value of NLR. [10].

While there have been many reports of venous thromboembolism in patients with COVID-19, literature on arterial thrombosis is limited [11,12,13]. Acute limb ischemia (ALI) is associated with blood hypercoagulability and can have either embolic or thrombotic causes [14].

Clinical manifestations of acute limb ischemia are the six Ps: pain, pulse deficit, pallor, paresthesia, paralysis, poikilothermia [15]. The incidence of ALI is approximately 1.5 cases out of 10,000 people per year [16]. Complications among ALI patients are high, and despite early revascularization, 30-day mortality and amputation rates are between 10% and 15% [17].

This retrospective study’s intentions were to report the incidence of arterial thrombosis of lower extremities and their complications in the acute phase of the infection among COVID-19 patients admitted to the hospital for treatment.

## 2. Materials and Methods

This was a retrospective study approved by the Hospital Ethics Committee (MS-20-02). In total, eighteen patients (*n* = 18) with arterial thrombosis were included in this retrospective study, all of whom were admitted via the Emergency unit of Infectology Clinic at University Hospital of Split (UH Split) in Croatia with a COVID-19 infection diagnosed prior to admission. Patients were selected from the cohort of 4762 patients treated for a COVID-19 infection and related complications within the UH Split from 19 March 2020 to 10 December 2021. Patients were selected with the inclusion criteria: (i) an RT-PCR (reverse transcription-polymerase chain reaction) confirmed SARS-CoV-2 infection at triage examination, (ii) patients admitted for treatment for COVID-19 disease in the UH Split, and (iii) an acute arterial thrombosis, confirmed by (CTA) computed tomography angiography. Patients were excluded from the study if they were PCR-negative with symptoms prolonged over acute COVID-19 disease or were previously treated outside UH Split. One out of eighteen patients was excluded due to severe COVID-19-related pneumonia and kidney failure, so intervention was not performed owing to his moribund condition.

All patients involved underwent CTA on a 128-layer Philips MRC 880. CTA imaging included a first unenhanced display of the peripheral arteries to clearly visualise calcifications. After that, an arterial imaging phase was performed in a thin layer (0.625–1.25) from the base of the lungs to the feet with a bolus placed in the aorta (at 150 HU), at the level of the L1 vertebral trunk. Immediately after that, we repeated the mid-thigh-to-foot scan to rule out unpacified blood vessels because the scanner crosses the bolus. All patients received 125 mL of iodine contrast agent (ioheksol 350 mg ioda/mL (Omnipaque 350, GE Healthcare)) at a rate of 4 mL/s. Multiplanar and VRT reconstructions were performed and read by radiologists with 12 years of experience in radiological angiology.

By analysing the data from CTA, we divided the percentage of occlusion of vessels by subocclusion or complete occlusion. Moreso, we summed thrombus lengths in patients who had several consecutive thrombi and viewed the sum as a single thrombus.

Medical history, medications, coagulation, and inflammatory laboratory data, interventions, and outcomes were reviewed from the patient’s charts. The kits used for the laboratory findings were as follows: for D-dimer (Innovance D-dimer Siemens, Siemens BCS, Erlangen, Federal Republic of Germany) with a referent value < 0.50 mg/L; for %PT—the prothrombin time activity percentage (Innovine PT, Siemens BCS, Erlangen Federal Republic of Germany) with a referent value > 0.70; for high sensitivity troponin (hsTroponin T) (Roche ECLIA, Cobas 6000, Roche Diagnostics International AG, Rotkreuz, Switzerland), with a referent value < 14 ng/L; and for N-terminal–pro-brain natriuretic peptide (NT-proBNP) (Roche ECLIA, Cobas 6000, Roche Diagnostics International AG, Rotkreuz, Switzerland), pg/mL. Referent values for NTproBNP varied depending on age and gender: male (55–64 years) < 386 pg/mL, (>65 years) < 879 pg/mL; women (55–64 years) < 352 pg/mL; (>65 years) < 624 pg/mL.

The prothrombin time activity percentage 70–120- in our laboratory-obtained values is divided by 100, so the normal values are 0.7–1.2 and are denoted as >0.7.

Prothrombin time (PT) results can be reported as clotting time (in seconds-PTs), activity percentage (%PT), ratio (PTr), and international normalised ratio (INR). In the University Hospital of Split, results of prothrombin time are expressed in terms of prothrombin time activity percentage (“activity percentage” divided by 100). The normal reference value of PT activity percentage is 70–120%.

### Statistical Analysis

Data were presented in descriptive statistics, with mean, standard deviation, median, minimum, and maximum for quantitative variables, while the frequency and percentage described qualitative variables. Thrombi were described by length and the degree of vessel stenosis. Thrombi were divided into the short segment (<10 cm), intermediate segment (10–20 cm), and long segment (>20 cm) occlusions [18]. Thrombi of adjacent arteries were considered as one unit. Moreover, we divided thrombi based on subocclusions and the artery’s total occlusion (100%). Fisher’s exact test was used to determine the association between the presence of COVID-19 pneumonia and outcome (death or live outcome), while the difference in haematological, biochemical, and CTA parameters between the COVID-19 diagnosed patients with X-ray-confirmed and non-confirmed pneumonia were assessed by t-test. To confirm the potential influence of predictor variables (haematological parameters, thrombus length parameters, blood vessel occlusion parameters expressed in percentages) to the results of X-ray, multiple regression analysis, and a general regression model were used. Statistical significance in all used methods was reduced to <0.05. The STATISTICA 11.0 software package was used for statistical data processing.

## 3. Results

### Clinical Data

Demographic and clinical characteristics of study participants are presented in Table 1. As can be seen from Table 1, patients were predominantly males (64.7%), with a median age of 75 years, and blood group type A (58.8%). The most common comorbidity was arterial hypertension (50.0%), while other less frequent comorbidities were diabetes mellitus type 2, hypothyroidism, atrial fibrillation, and rheumatoid arthritis. All of them presented without AT symptoms on hospital admission. In total, three patients (16.7%) reported treatments of comorbidities with anticoagulant therapy. None of the seventeen patients had previous signs or symptoms of AT.

Most of the patients were admitted to the Infectology Clinic on the seventh day of COVID-19 disease with COVID-19 related symptoms, such as prolonged temperature, fatigue, myalgia, and shortness of breath. COVID-19 pneumonia was diagnosed in 13 patients by chest X-ray imaging. On the tenth day, from the onset of the disease, patients developed symptoms related to acute limb ischemia (pain, pallor skin, distal pulse deficit of lower extremities, paresthesia) observed by physical exams. Patients underwent CT angiography imaging and were diagnosed with peripheral AT, and laboratory tests were performed on the same day (Table 2). Out of 13 patients with COVID-19 pneumonia, 10 (76.9%) developed acute respiratory failure, and 7 (53.8%) required oxygen therapy via a high-flow nasal cannula (HFNC) system. Statistical analysis showed a significant relationship between patients with COVID-19 pneumonia and survival outcome (*p* = 0.011). Laboratory findings in patients with X-ray confirmed pneumonia showed four times higher the neutrophil-to-lymphocyte ratio (NLR; *p* = 0.035) and C-reactive protein (CRP; *p* = 0.016), and three times higher lactate dehydrogenase levels (LDH; *p* = 0.038) than patients without confirmed pneumonia (Table 2). Creatine kinase was almost four times higher in patients with pneumonia than without. Results of multiple regression analysis of the influence of predictor variables (laboratory results) on lung X-ray results in COVID-19 positive patients showed a statistically significant influence (*p* = 0.040) of the parameters falling in the next series of CRP (*p* = 0.010) > LDH (*p* = 0.035) > NLR (*p* = 0.0483).

#### Parameters Related to Arterial Thrombosis

**Figure 1:**
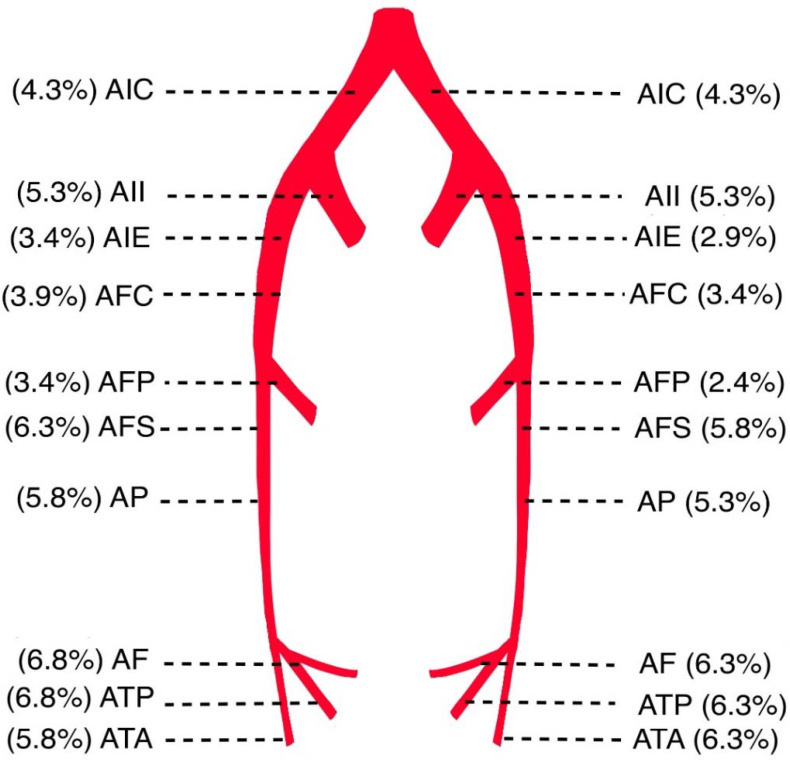
Frequency of arteries affected by arterial thrombosis in patients of the study population. AIC: a.iliaca communis; AII: a.iliaca interna; AIE: a.iliaca externa; AFC: a.femoralis communis; AFP: a.femoralis profunda; AFS: a.femoralis superficialis; AP: a.poplitea; ATA: a.tibialis anterior; AF: a.fibularis; ATP: a.tibialis posterior.

Sixteen patients (94.1%) developed bilateral arterial occlusions, with one showing occlusion of one limb (Table 3). Most often, the occlusions were in the lower half of the lower extremities. The frequency of arteries affected by arterial thrombosis in patients of the study population is shown in Figure 1. Patients with pneumonia had significantly more bilateral occlusions of the lower extremities and a significantly higher percentage with complete occlusion of the arteries than patients without pneumonia.

**Figure 2:**
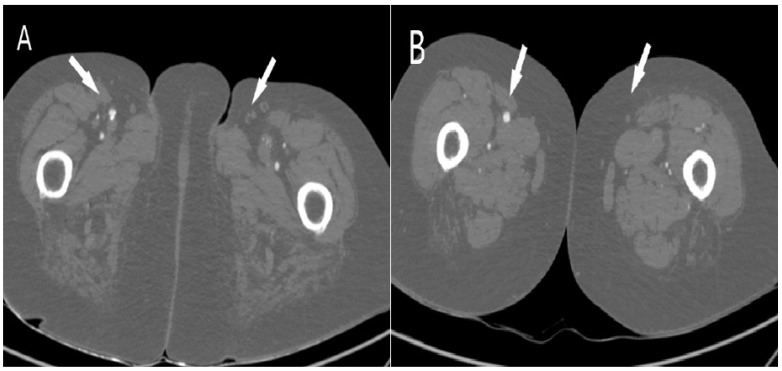
Axial computed tomography angiography shows (**A**) occlusion of the left common femoral artery (white arrow) and (**B**) shows occlusion of the left superficial femoral artery (white arrow).

**Figure 3:**
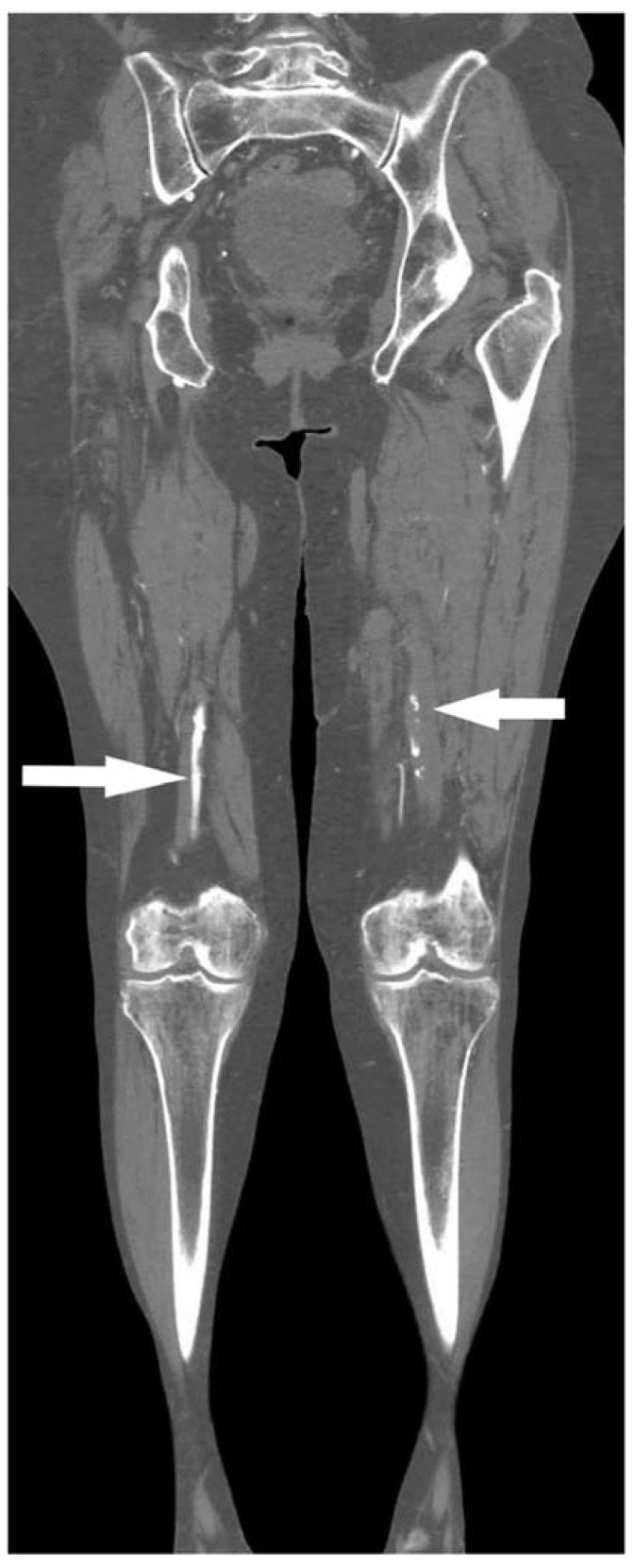
Coronal computed tomography angiography shows occlusion of the left common femoral artery (white arrows), the same patient as in Figure 2.

**Figure 4:**
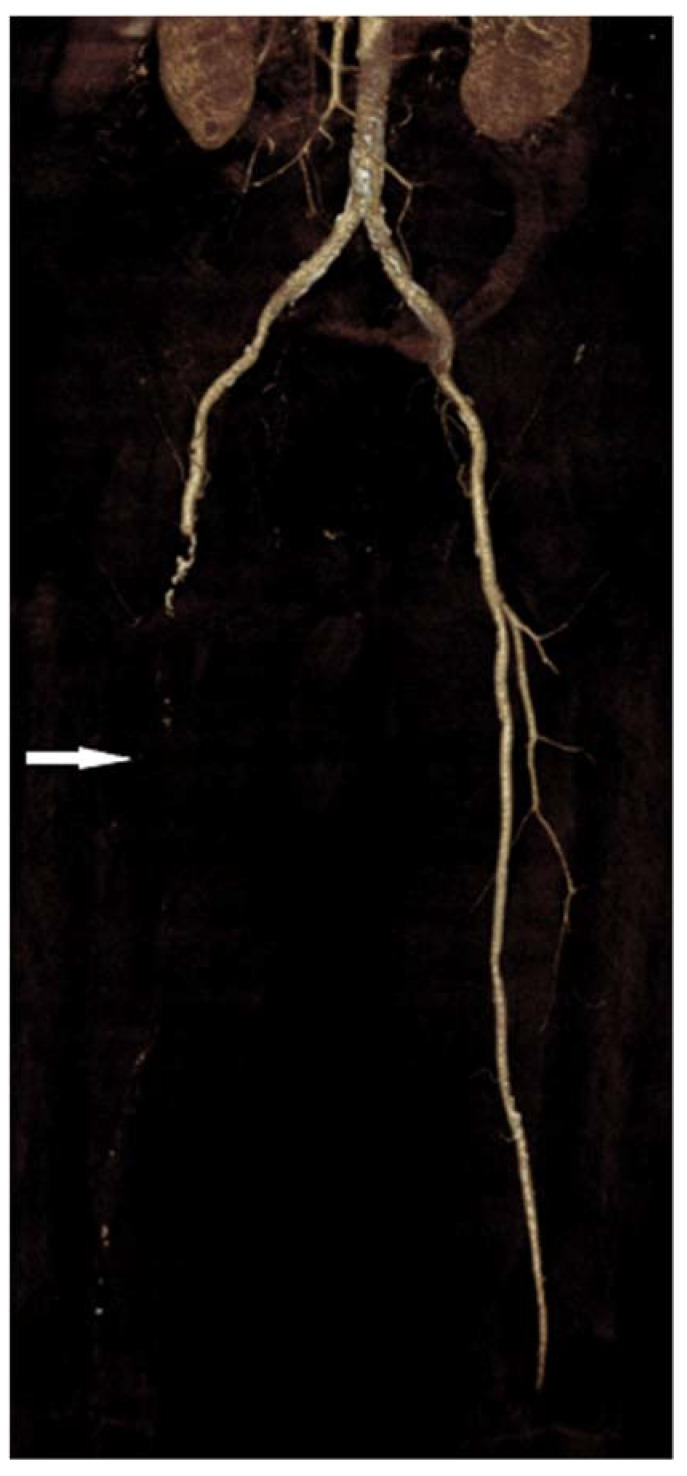
CTA volume-rendered 3D reconstruction from a 66-year-old male demonstrates long segment right superficial femoral artery occlusion (white arrow).

Observing the length and stenosis degree, most thrombi were long segments (48.7%), causing total vessel occlusion (37.9%), as shown in Table 3. There were three times more total occlusions of arterial vessels (*n* = 59) in patients with pneumonia than in patients without COVID-19 pneumonia (*n* = 18). The t-test did not confirm a statistically significant difference between the mean thrombus length values obtained by CTA screening between patients diagnosed with COVID-19 with X-ray-confirmed and unconfirmed pneumonia. Multiple regression analysis showed a very weak but not statistically significant (R = 0.24; *p* = 0.8714) correlation between predictor variables (percentage of blood vessel occlusion) and lung X-ray results. Before surgical treatment of arterial thrombosis, patients received prophylaxis doses of low molecular weight heparins (LMWH), with three patients having therapeutic doses. Fifty percent of patients underwent thrombectomy, while limb amputation was indicated in six patients. However, due to the severity of COVID-19 infection and arteriothrombotic complications, overall, ten patients (58.8%) died during a hospital stay. All of them had COVID-19 pneumonia. In a group of patients without pneumonia, all patients were discharged. Figure 2, Figure 3 and Figure 4 present CTA images of one of the patients from our cohort.

## 4. Discussion

The viral coronavirus disease of 2019 (COVID-19), predominantly affecting the respiratory system, also increases the risk of thromboembolic incidents due to COVID-19 induced coagulopathy, with higher mortality rates [19]. In addition, novel reports from the pandemic observed an abnormal coagulation function in patients with COVID-19 pneumonia, with findings of elevated D-dimer and fibrin degradation products [7,20,21]. The underlying mechanisms of such events are still not completely understood. Still, they may include a hypercoagulability state, inflammation and cytokine storm, endothelial dysfunction, and an aberrant renin-angiotensin-aldosterone (RAAS) axis due to the binding of SARS-CoV-2 to the endothelial ACE-2. The binding of SARS-CoV-2 to ACE-2 is a crucial element for viral infectivity and multi-organ damage. ACE-2 is expressed in various human tissues, such as the CNS, skeletal muscle, gastrointestinal tract, and endothelial cells [22].

A sudden and significant increase of COVID-19–infected patients who were presenting with ALI has been noted at our institution since the pandemic. A similar increase in the incidence rate of patients presenting with ALI in 2019 occurred compared with 2020 (1.8% vs. 16.3%) and was reported by Bellosta et al. [21].

Several mechanisms have been proposed to explain the high incidence of thrombotic events during COVID-19 infection. The normal physiological endothelial function refers to the ability to regulate vascular tonus, permeability, cell adhesion, and anticoagulation. Healthy endothelial cells synthesise nitric oxide (NO) by conversion of l-arginine to l-citrulline by nitric oxide synthase. The NO released by the endothelium prevents leukocyte and platelet adhesion, inflammatory cell migration into the vessel wall, smooth muscle cell proliferation, and suppresses apoptosis and inflammation. SARS-CoV-2 enters endothelial cells through endocytosis and is mediated by an interplay of angiotensin-converting enzyme 2 (ACE-2) and the transmembrane protease serine 2 (TMPRSS-2) which sheds a part of the spike protein and helps SARS-CoV-2 enter into the endothelial cells. The infected endothelial cells lose their ability to maintain the aforementioned physiological functions. Subsequently, the damage of the endothelium leads to the procoagulant change of the vascular lumen, a formation of immunothrombosis, and organ malcirculation [23].

Ali and Spinler, in their study [19], reported the potential mechanisms of COVID-19 induced thrombosis. They dysregulated the renin-angiotensin-aldosterone system and the role of ACE2, causing oxidative stress damage, endothelial dysfunction, and the activation of the von Willebrand factor, and dysregulated the immune response, role of the complement system, neutrophil extracellular traps (NETs), and mitogen-activated protein kinases (MAPKs) pathways.

In this retrospective study, we reported 17 patients who were admitted to the UH Split due to the severity of COVID-19 infection. Fourteen patients presented with COVID-19 pneumonia, diagnosed with a chest X-ray. However, with the latency of a median 2-day difference from the admission day, patients developed ALI symptoms, which were further examined. They were diagnosed with acute, native thromboembolic events of the aorta and lower limb arteries by CTA. A retrospective study by Cantador et al. reported data about acute cerebrovascular events in COVID-19 and included 214 patients, with about 6% presenting with acute cerebrovascular events, mainly ischemic strokes. Stroke symptoms tend to appear later during hospitalisation, a median of 10 days after the onset of symptoms [24]. In COVID-19, both alveolar damage and a microcirculatory disturbance associated with thrombus formation contribute to respiratory dysfunction. 

Laboratory findings in patients of this report showed mixed results in a hypercoagulable state. Prothrombin time ratios were relatively within typical range values, with elevated D-dimer values in all patients but without a significant difference between groups. However, inflammation parameters (CRP, lymphopenia), LDH—as a tissue injury parameter—and CK were significantly elevated in patients with COVID-19 pneumonia, contrary to normal CXR findings. In addition, previous studies reported that the neutrophile-to-lymphocyte ratio (NLR) was an independent risk factor for COVID-19 severity [25,26], which we observed to be significantly higher in patients with COVID-19 pneumonia.

Demographic data were in accordance with other similar studies: male patients, around 75 years with comorbidities, but without a history of arterial thrombosis, dominated [21,27].

Analysing MCST data showed mostly bilateral arterial occlusions in the lower half of the lower extremities. In the group of 17 studied patients, there were 77 total occlusions (100%) of the arterials at the lower extremities, especially in the group with COVID-19 pneumonia (*n* = 59), which presented a very high number. Italian authors revealed data in their study [21], those arterial thrombi in their COVID-19 patients, even macroscopically, appear quite different from specimens before COVID-19.

The most affected arteries were a.fibularis (AF) and a.tibialis posterior (ATP). Etkin et al., in their study, conducted in New York State, reported that 71% of the arterial occlusions were in the lower extremities, particularly below the knee (43%) [28]. Similar data were presented by Singh et al. in their study [29].

The rate of limb loss was 35.3%. They were all from the group with COVID-19 pneumonia. An American study [28] reported that out of the 35 patients with lower extremity ischemia, 5 patients (14.2 %) had primary amputations.

Unfortunately, the rate of mortality was 58.8%. Similar results were reported by Etkin et al. [28], while the study by Cheruiyot et al. (19%) and Bellosta et al. reported lower mortality rates (40%) [21,27]. Ten out of thirteen patients with pneumonia died due to acute respiratory distress syndrome (ARDS). All patients without pneumonia were discharged from the hospital.

Since the acute arterial ischemic events were noted as the presenting symptom in an increasing number of patients, a diagnosis of COVID-19 should be considered in any patient presenting with arterial ischemia. In addition, our data suggest that native arterial thrombosis might be triggered by COVID-19 infection.

### Study Limitations

This retrospective study has major limitations. First, we included a small cohort of 17 patients in the study. Moreso, this is a retrospective descriptive review that did not include a comparison with patients without COVID-19 infection (control group). With the small number of patients, it is difficult to estimate the risk factors for illness distribution and outcomes. Lastly, the actual incidence of acute arterial limb ischemic events in patients with COVID-19 is not complete due to many patients without symptoms of ALI or in a moribund state.

## 5. Conclusions

The results of this study support new findings on the effects of COVID-19 disease on the cardiovascular system. Arterial thrombosis with consequent lower limb ischemia, resulting in amputations and death, is more common in patients with COVID-19 with severe pneumonia. Therefore, a SARS-CoV-2 positive PCR test, in combination with symptoms, significantly elevated the laboratory parameters (CRP, NLR, LDH) and radiological findings of pneumonia, and may refer to patients at risk of developing arterial thrombosis, and thus, urgent CTA diagnosis is necessary.

## Figures and Tables

**Table 1:** Demographic characteristics of the patients.

Total No. of Patients	*n* = 17
Age, years (median)	75.0 (61–90)
Gender, *n* (%)	
Male	11 (64.7)
Female	6 (35.3)
Blood type, *n* (%)	
O	5 (29.4)
A	10 (58.8)
AB	2 (11.8)
Comorbidities, *n* (%)	
Arterial hypertension	10 (50.0)
Diabetes mellitus type 2	3 (15.5)
Hypothyroidism	3 (15.5)
Atrial fibrillation	3 (15.5)
Rheumatoid arthritis	1 (5.0)
COVID-19 day at admission	
Mean (SD)	7.65 (3.45)
Arteriothrombosis diagnosis day	
Mean (SD)	9.76 (2.68)
History of anticoagulant therapy, *n* (%)	3 (16.7)
Patients with acute respiratory failure, *n* (%)	10 (58.8)
Patients receiving oxygen therapy, *n* (%)	12 (70.5)
Patients with oxygen therapy via HFNC, *n* (%)	7 (41.2)

*n*: number of patients; %: percentage; SD: standard deviation; IQR: interquartile range; HFNC: high-flow nasal cannula.

**Table 2:** Blood parameters on the day of arterial thrombosis signs and symptoms appearing in patients with/without COVID-19 pneumonia.

Characteristics, Mean (SD)	Patients Values	Patients with Pneumonia	Patients without Pneumonia	*p*
Neutrophils, ×10^9^/L	14.22 (6.72)	15.88 (6.19)	8.39 (5.67)	0.025 *
Lymphocytes, ×10^9^/L	0.98 (0.55)	0.88 (0.51)	1.35 (0.55)	0.018 *
LDH U/L	725.6 (532.9)	841.0 (552.7)	321.8 (79.9)	0.038 *
CRP, mg/L	92.4 (78.7)	111.0 (79.2)	27.4 (25.4)	0.016 *
D-dimer mg/L	22.11 (11.08)	24.78 (9.12)	12.73 (13.59)	0.170
Thrombocytes ×10^9^/L	305.78 (114.67)	297.07 (107.98)	336.25 (149.61)	0.611
Creatine kinase U/L	1301.28 (1356.16)	1554.57 (1421.12)	415.75 (568.07)	0.031 *
NT-proBNP pg/mL	1849.94 (2104.41)	2042.71 (2361.44)	1175.25 (387.48)	0.556
Troponin ng/L	91.63 (129.25)	96.22 (144.88)	75.55 (57.04)	0.758
Neutrophils-lymphocyte ratio (NLR)	22.4 (20.1)	27.0 (20.5)	6.1 (2.2)	0.035 *
%PT	73 (22)	72 (22)	78 (22)	0.555
INR	1.22 (0.12)	1.23 (1.13)	1.19 (0.11)	0.875

*: statistically significant; SD: standard deviation; *p*: *p*-value; CRP: C-reactive protein; LDH: lactate-dehydrogenase; NT-proBNP: N-terminal–pro-brain natriuretic peptide; %PT: prothrombin time activity percentage; INR: international normalised ratio.

**Table 3:** CTA findings, intervention, and outcome data of the patients.

	All Patients	Patients with Pneumonia	Patients without Pneumonia
	*n* = 17	*n* = 13	*n* = 4
Patients with unilateral occlusions, *n* (%)	1 (5.9)	1 (5.9)	0
Patients with bilateral occlusions, *n* (%)	16 (94.1)	12 (70.6)	4 (23.5)
Occlusions by stenosis degree, *n* (%)			
Subocclusions	126 (62.1)	100 (49.3)	26 (12.8)
Total occlusions (100%)	77 (37.9)	59 (29.1)	18 (8.8)
Thrombus length, *n* (%) ^b^			
Short segment occlusions (<10 cm)	36 (32.4)	34 (30.6)	2 (1.8)
Intermediate segment occlusions (10–20 cm)	21 (18.9)	13 (11.7)	8 (7.2)
Long segment occlusions (>20 cm)	54 (48.7)	39 (35.1)	15 (13.5)
Anticoagulant therapy before surgery, *n* (%) ^a^			
Prophylaxis dose	14 (82.4)	11 (64.7)	3 (17.6)
Therapeutic dose	3 (17.6)	2 (11.8)	1 (5.9)
Intervention, *n* (%)			
Conservative (LMWH)	2 (11.8)	1 (5.9)	1 (5.9)
Thrombectomy	9 (52.9)	6 (35.3)	3 (17.6)
Amputation	6 (35.3)	6 (35.3)	0
Outcome, *n* (%)			
Death	10 (58.8)	10 (58.8)	0
Discharge	7 (41.2)	3 (17.6)	4 (23.5)
CXR chest X-ray, LMWH low molecular weight heparin.			

^a^ Number of affected patients divided by the total patient number (*n* = 17). ^b^ Number of observations is divided by the total count of occlusions (*n* = 203) or thrombus (*n* = 111).

## Data Availability

Data are available from the digital records of the University Hospital of Split.
